# Extracellular pH is a biomarker enabling detection of breast cancer and liver cancer using CEST MRI

**DOI:** 10.18632/oncotarget.17404

**Published:** 2017-04-25

**Authors:** Miaomiao Chen, Chaoying Chen, Zhiwei Shen, Xiaolei Zhang, Yanzi Chen, Fengfeng Lin, Xilun Ma, Caiyu Zhuang, Yifei Mao, Haochuan Gan, Peidong Chen, Xiaodan Zong, Renhua Wu

**Affiliations:** ^1^ Department of Medical Imaging, The Second Affiliated Hospital, Medical College of Shantou University, Shantou 515041, China; ^2^ Department of Clinical Laboratory, The First Affiliated Hospital of Hunan Traditional Chinese Medical College, Zhuzhou 412000, China; ^3^ Provincial Key Laboratory of Medical Molecular Imaging, Shantou 515041, China

**Keywords:** liver cancer, pH imaging, breast cancer, ioversol, CEST MRI

## Abstract

Extracellular pH (pH_e_) decrease is associated with tumor growth, invasion, metastasis, and chemoresistance, which can be detected by chemical exchange saturation transfer (CEST) magnetic resonance imaging (MRI). Here, we demonstrated that ioversol CEST MRI can be exploited to achieve pH_e_ mapping of the liver cancer microenvironment. In *in vitro* studies, we firstly explored whether ioversol signal is pH-dependent, and calculated the function equation between the CEST effects of ioversol and pH values, in the range of 6.0 to 7.8, by a ratiometric method. Then we verified the feasibility of this technique and the equation *in vivo* by applying pH_e_ imaging in an MMTV-Erbb2 transgenic mouse breast cancer model, which is often used in CEST pH_e_ studies. Furthermore, *in vivo* ioversol CEST MRI, we were able to map relative pH_e_ and differentiate between tumor and normal tissue in a McA-RH7777 rat hepatoma model. This suggests pH_e_ may be a useful biomarker for human liver cancer.

## INTRODUCTION

Cancer remains one of the most difficult diseases to cure, with successful treatment primarily relying on early diagnosis. However, the clinical outcome is often insufficient because the patients’ quality of life is already poor when they are diagnosed. Thus, clinical success would be greatly enhanced by a noninvasive method for identification of malignant transformation. As reported in recent decades, a reduction in extracellular pH (pH_e_) is a characteristic of most malignant tumors [1]. The pH_e_ of the tumor microenvironment is typically acidic, in the range of 6.5 to 6.9, whereas the pH_e_ of normal tissue is approximately 7.2 to 7.5 [2]. Extracellular tumor acidosis is the result of increased lactic acid production by high aerobic glycolysis [3] and poor perfusion of tumor cells [4]. This resultant acidity can enhance tumor aggressiveness [5], metastasis [6], chromosomal rearrangements [7], and angiogenesis [8]. Furthermore, low pH_e_ also leads to resistance to radiation treatment and specific chemotherapeutics [9]. Acidic tumors are more sensitive to weak-acid chemotherapies such as esomeprazole than to weak-base chemotherapies such as doxorubicin [10–12]. Additionally, modulation of tumor pH_e_ in a breast cancer mouse model has been shown to reduce metastasis and improve survival [13]. Given the association of acidity with tumor malignancy, accurate measurement of tumor pH_e_ may greatly aid in identification of malignancy and tumor invasion, as well as in predicting therapeutic efficacy.

**Figure 1:**
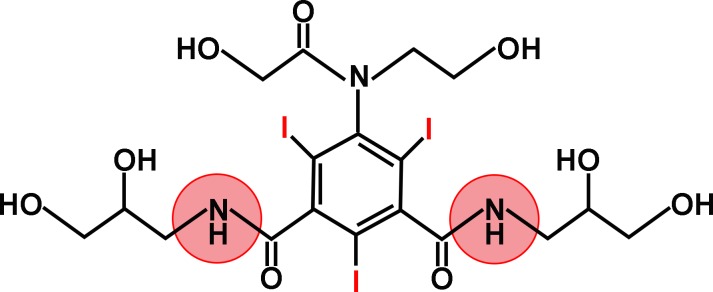
Ioversol chemical structure Ioversol contains 2 amide groups, 4.3 ppm downfield from the bulk water resonance.

A variety of biomedical imaging methods have been developed to monitor tumor pH_e_ *in vivo*. Optical imaging is a low cost imaging tool with good sensitivity, but can only evaluate surface-accessible tumors [14]. Whole-body PET allows rapid measurement of pH_e_ [15]. Magnetic resonance spectroscopy provides excellent contrast with limited radiation exposure. However, both methods suffer from limited accuracy, poor sensitivity, low spatial resolution, and limited diagnostic ability [16, 17]. Based on these limitations, a novel magnetic resonance imaging contrast mechanism called chemical exchange saturation transfer may be desirable. Unlike traditional MRI, which is operated by altering the T_1_ or T_2_ relaxation times of water protons, CEST effects are detected indirectly through the reduced water signal obtained after selectively saturating the exchangeable protons on a CEST agent using radiofrequency (RF) irradiation [18–21]. CEST contrast agents map the pH_e_ in the microenvironment in which they distribute [22]. Generally, CEST effects are modulated by several factors including pH, contrast agent concentration, and temperature [23, 24]. In order to minimize the effect of contrast agent concentration, ratiometric methods have been introduced [25]. Several iodinated contrast agents have been employed to measure tissue pH_e_ [26, 27]. Ioversol, a widely used non-ionic X-ray contrast agent with high water solubility and low toxicity, has been shown to be pH-dependent [26]. Ioversol has two amide groups detected 4.3 ppm downfield from water (Figure 1), and has different CEST effects at different RF power levels, allowing an RF power-based ratiometric method to rule out concentration effects [27], and making ioversol more sensitive than traditional iodinated contrast agents such as iopamidol. However, the correlation between the CEST effects of ioversol and pH value still remains unclear.

There are several reports concerning tumor CEST pH_e_ imaging, especially in breast cancer, indicating that CEST pH_e_ imaging can detect the acidic pH_e_ of tumors [4, 12, 27], which is helpful for identifying malignancy in the clinical works. However, for liver cancer, which is of high incidence [28], little is known about its pH_e_ imaging. Therefore, we investigated whether CEST MRI could be used as a noninvasive method to diagnose and analyze liver cancer. In this study, we predicted that liver cancer tissue would show a significantly lower CEST signal compared with normal liver tissue. Initially, we aimed to explore the correlation between CEST effects and pH values of ioversol *in vitro*, at high magnetic fields, and then applied our findings to a breast cancer model to demonstrate the feasibility of CEST imaging for liver cancer *in vivo*. Finally, we obtained a pH_e_ map of liver cancer, which provides a novel imaging method for liver cancer diagnosis in the clinical setting, and predicting the efficacy of clinical treatment [29].

## RESULTS

### CEST effects of ioversol

**Figure 2:**
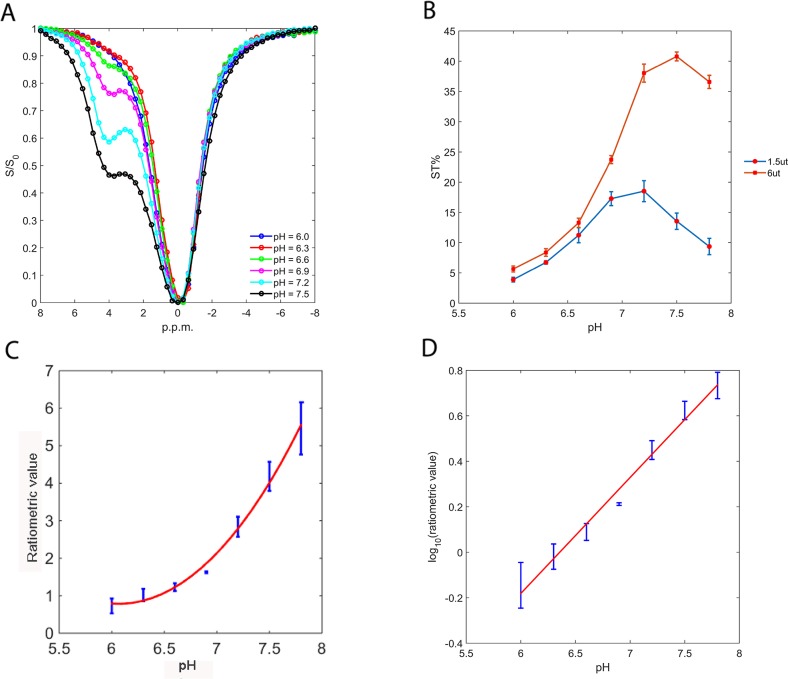
Ioversol exhibits a strong CEST signal **(A)** Z-spectra of 30 mM ioversol at different pH values at 6 μT. **(B)** Ioversol ST% depends on pH at RF saturation powers of 1.5 and 6 μT. **(C)** The CEST ratio was exponentially correlated with pH. **(D)** The log_10_ ratio of the CEST effect linearly correlated with pH.

As the CEST signals are pH-dependent, we first obtained the CEST Z-spectra of 30 mM ioversol at varying pH and under a B_1_ irradiation level of 6 μT (Figure 2A). The spectra revealed broad CEST effects, with a peak at 4.3 ppm corresponding to the two exchangeable amide groups. With increasing pH, the signals broadened because of the higher exchange rate. To evaluate the CEST effects, we measured the saturation transfer effect (ST%) within a pH range of 6.0 to 7.8 *in vitro*, using two saturation pulse powers (1.5 and 6 μT, Figure 2B). As expected, the CEST effects were markedly pH-dependent, with minimum values at pH 6.0, that gradually increased with pH up to pH 7.5 at an irradiation power of 6 μT, and to pH 7.2 at 1.5 μT, then decreased at higher pH. At high pH, the CEST effect decreases because the exchange rate becomes too high [26]. The unequal CEST effects under different irradiation powers enable a novel ratiometric pH MRI method first proposed by Longo et al [27].

Theratiometric value is calculated according to the following equation:

$$\text{RPM}=\frac{{\left[\left(1-\text{ST}\right)/\text{ST}\right]}_{\text{RF}1}}{{\left[\left(1-\text{ST}\right)/\text{ST}\right]}_{\text{RF}2}}$$

ST_RF1,2_ represents the saturation transfer (ST) acquired under different RF powers. Then we calculated the RF power mismatch (RPM) by determining the ST% ratio at RF power levels of 1.5 and 6 μT (Figure 2C). The RPM curve demonstrated that the ratiometric values increased exponentially with the pH value in the pH range of 6.0 to 7.8, with the log_10_ ratio of the CEST effects exhibiting an excellent correlation with pH (R^2^ = 0.98, *p* < 0.001, Figure 2D).

### *In vitro* imaging of ioversol

**Figure 3:**
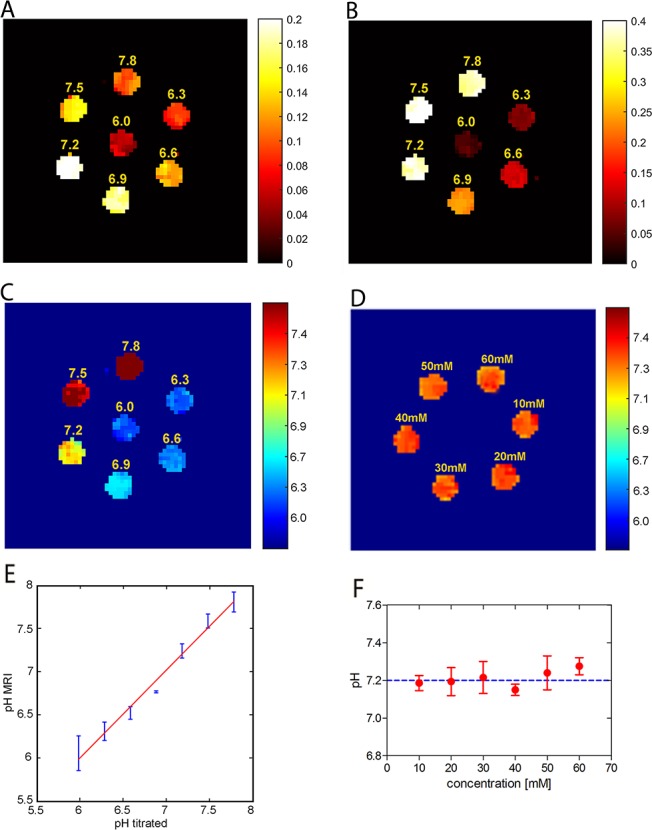
Ioversol CEST MR images of a phantom consisting of test tubes ST images of 30 mM ioversol at different pH values under RF powers of 1.5 μT **(A)** and 6 μT **(B)**. **(C)** pH map calculated using the corresponding ST images **(A and B)**. **(D)** pH mapping is independent of ioversol concentration. **(E)** The calculated pH values strongly correlate with the titrated pH values. **(F)** Mean pH values calculated for several concentrations.

Next, to determine whether the corresponding pH images could distinguish the ioversol solutions at different pH values, we performed experiments on 7 tubes of 30 mM ioversol at pH 6.0 to 7.8 and under B_1_ irradiation levels of 1.5 and 6 μT. Figure 3A and Figure 3B show strong CEST signal changes occurred with increasing pH, with the signal changes at 6 μT (Figure 3B) being greater than that at 1.5 μT (Figure 3A). Then we obtained a pH map by calculating from the ratiometric map, which was calculated from the ratio of the corresponding ST images (Figure 3C). The pH map showed that the different pH values could be clearly distinguished by the colorful signals. The calculated pH values strongly correlate with the titrated pH values, with pH_(MRI)_ = pH_(titrated)_ + 2E^−0.5^ (R^2^ = 0.98, *p* < 0.001) (Figure 3E). It should be noted that, this CEST agent has been previously used for *in vivo* applications, so we must ensure that it is concentration-independent. To answer this question, we prepared 6 tubes of pH 7.2 ioversol solution with different ioversol concentrations (10-60 mM). The pH map revealed no significant difference between these ioversol solutions (Figure 3D), and the calculated pH values were obtained within a small error for all concentrations (Figure 3F), confirming that pH quantification by ioversol CEST imaging can indeed measure pH over the 6-fold concentration range used in this study.

### *In vivo* imaging of breast cancer

**Figure 4:**
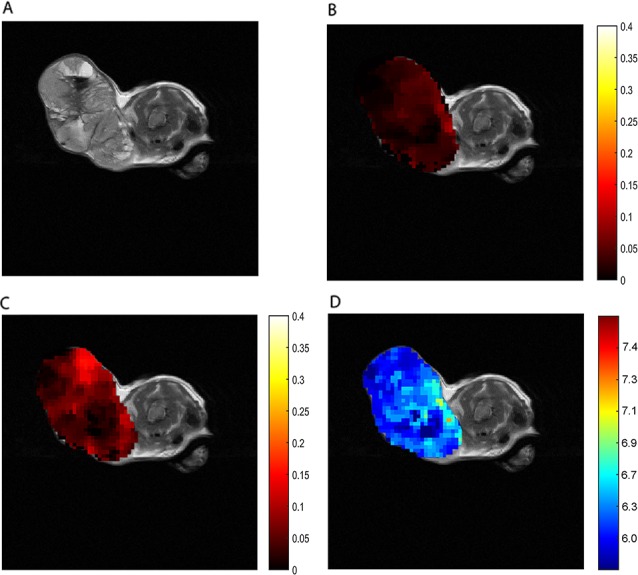
*In vivo* CEST imaging of breast cancer **(A)** T_2_ breast cancer image. ST map after ioversol injection at 1.5 μT **(B)** and 6 μT **(C)**. **(D)** The pH_e_ map calculated by using corresponding ST images **(B and C)**.

To assess whether our *in vitro* results are relevant for *in vivo* studies, we first applied ioversol CEST pH_e_ in themouse mammary tumor virus (MMTV)-*Erbb2* transgenic mouse breast tumor model, which is often used in CEST pH_e_ studies. The axial T_2_ image of a breast cancer before injection shows the location of the tumor (Figure 4A). Then we directly injected ioversol into the tail vein, whereupon a strong CEST signal was generated in the tumor. The CEST ST maps, from the tumor region at 1.5 μT (Figure 4B) and 6 μT (Figure 4C) power saturation, showed that at 6 μT in the tumor (Figure 4C) was brighter than at 1.5 μT (Figure 4B), owing to the faster exchange rate. The tumor pH_e_ map (ratiometric map in Figure 4B and 4C) of the tumor superimposed on the anatomic MR image shows an acidic extracellular environment, in the tumor region, corresponding to pH_e_ values between 6.3-6.9 (Figure 4D).

### *In vivo* imaging of normal liver and liver tumors

**Figure 5:**
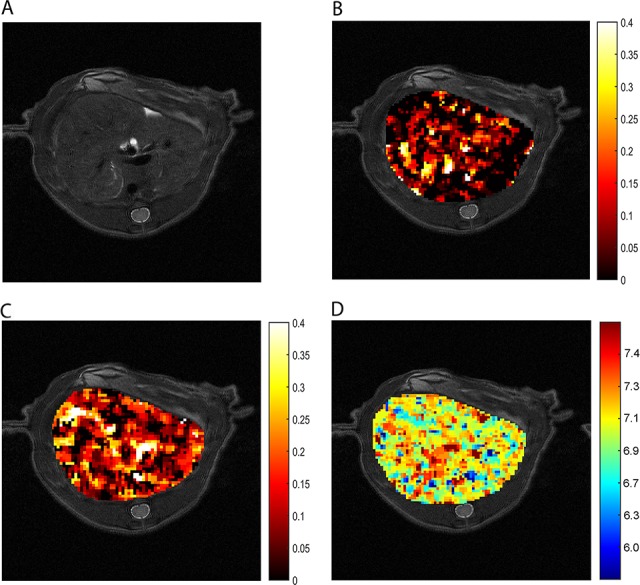
CEST imaging of normal liver tissue **(A)** T_2_ liver image. ST map after ioversol injection at 1.5 μT **(B)** and 6 μT **(C)**. **(D)** The pH_e_ map calculated using the corresponding ST images **(B and C)**.

**Figure 6:**
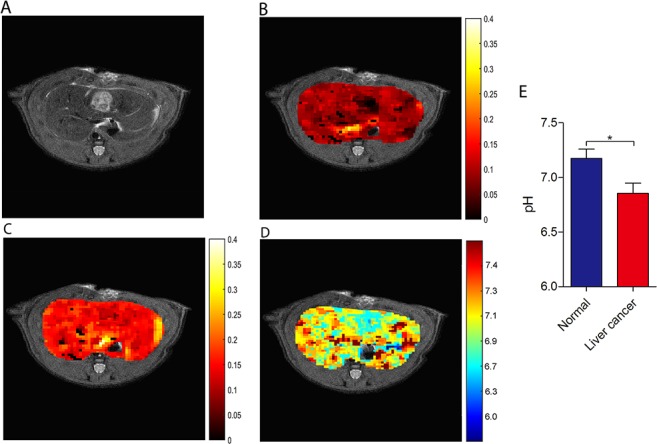
CEST imaging of liver cancer **(A)** T_2_ liver cancer image. ST map after ioversol injection at 1.5 μT **(B)** and 6 μT **(C)**. **(D)** The pH_e_ map calculated using corresponding ST images **(B and C)**. **(E)** pH values of the two tissues showing a significant difference (*p < 0.05, Student's *t*-test). Error bars represent the standard deviation (n = 10)

Next, we tested whether ioversol CEST imaging could be performed on liver tissue. A reasonable CEST signal was observed throughout the normal liver at RF powers of 1.5 and 6 μT (Figure 5B and 5C, respectively), and the signal of the calculated pH_e_ map (Figure 5D) displayed well in the region of the liver on the T_2_ image (Figure 5A), indicating ioversol CEST pH_e_ MRI is feasible for liver applications. In an effort to determine whether ioversol CEST imaging could differentiate between liver tumor and normal tissue, we transplanted McA-RH7777 rat hepatoma cells into the left lateral hepatic lobe of Sprague-Dawley (SD) rats and allowed tumors to form for 14 days prior to ioversol CEST MRI. The axial T_2_ image showed that the tumor diameter was 9 mm (Figure 6A). After injecting ioversol, the liver tumor exhibited a lower CEST signal compared with the normal liver tissue, at both 1.5 μT (Figure 6B) and 6 μT (Figure 6C) irradiation. The ioversol CEST pH_e_ maps were obtained from the rat tumors at 7T (Figure 6D). The pH_e_ map clearly distinguished tumor from normal tissue, and the pH_e_ of the tumor was lower than that of normal liver tissue in all 10 rats examined (*p* < 0.05, Figure 5E). Interestingly, we also found in the ioversol CEST pH_e_ image that the tumor region was slightly greater than the region obtained by T_2_ imaging in both a breast and liver tumor model. Images of the tissue stained with hematoxylin and eosin (HE) are shown in the Supplementary Figure 1.

## DISCUSSION

Our findings provide the first evidence that ioversol CEST pH_e_ MRI can accurately measure liver and breast tumor pH_e_ in a concentration-independent manner, with excellent spatial resolution. In phantom studies, we showed that the log_10_ ratio of the CEST effect was linearly proportional to the pH value in the range of 6.0-7.8. These results extend the study of *Longo* et al., who only showed that ioversol is pH-dependent [26]. Moreover, compared with traditional CEST agents that have two CEST effects in one Z-spectrum, ioversol has two Z-spectra that may be more sensitive because the two CEST effects from different RF levels do not interact with each other. However, besides the contrast agent concentration, the CEST effect also depends on temperature, so attention must be paid to pH measurements at the same temperature during experiments [30].

Compared with the mouse xenograft models in previous reports, we choose the MMTV-*Erbb2* transgenic mouse breast tumor model for our study because this reflects human breast cancer characteristics more accurately [31]. As we can see from the results, the pH_e_ of the tumor region was fairly acidic, in the range of 6.3-6.9, suggesting a high degree of malignancy. We further show that liver cancer tissue had lower CEST signals compared with normal tissue. For our liver tumor model, we chose an orthotopic transplantation tumor model, using McA-RH7777 rat hepatoma cells, instead of a subcutaneous tumor model, because it simulates the characteristics and environment of human liver cancer. However, the accompanying challenges could not be neglected, especially motion artefacts and field inhomogeneity corrections. To minimize motion artifacts from breathing, we used a respiratory gating device and bellyband to reduce the respiratory amplitude. We also adopted motion correction and B_0_-shift correction to improve the image quality. In addition, a duration of 15 minutes post injection was allowed, to permit stabilization and prolonged accumulation of the contrast agent within the tumor, during the CEST MRI acquisition. However, the signal-to-noise ratio (SNR) in the liver of liver and liver cancer images was not always satisfactory, compared with kidney pH maps previously reported [25, 32]. This is mainly because: 1) the extracellular concentration of agent that accumulates in the liver is lower than that in the kidney [33, 34], and 2) the effect of breathing motion more profoundly affects the liver compared with that to kidney. The pH_e_ map of the tumor region was slightly larger than that of the T_2_ map, indicating that either the acid diffused out of the tumor, or the pH_e_ map was affected by noise, remaining to be further elucidated.

Future studies will be needed to optimize the parameters of this approach, for purpose of improving the image SNR. Shortening the acquisition time while maintaining good SNR by reducing the offset number also needs further investigation [35]. Moreover, as more and more patients accept chemotherapy, we should further explore and use CEST pH MRI to assess the response of liver cancer following chemotherapy in mouse models, and now we have compounded a nano-targeted chemotherapy medicine. Additionally, we will also attempt to translate this technique from high field to lower field strengths. It is important to note that we are preparing to explore ioversol pH imaging using a 3T MR scanner, as pH_e_ imaging with iodinated agents and CEST liver imaging in patients has been recently reported [35, 36]. Future studies using this approach may provide new insights into liver cancer development and biomarkers for the clinical studies.

## MATERIALS AND METHODS

### Phantom preparation

One phantom contained seven micro-centrifuge tubes with 30 mM ioversol (Hengrui Medicine Co., Ltd., Jiangsu, China) in phosphate-buffered solution, titrated using HCl or NaOH, to various pH values ranging from 6.0 to 7.8 in units of 0.3. The other phantom contained six micro-centrifuge tubes of pH 7.2 ioversol, at concentrations from 10 to 60 mM, in phosphate-buffered solution. The phantoms were filled with agarose solution and solidified at 37°C.

### Cell culture

The McA-RH7777 rat hepatoma cell line was purchased from the Chinese Academy of Sciences (Shanghai, China). Cells were cultured in Dulbecco's modified Eagle's medium (DMEM), supplemented with 10% fetal bovine serum and 2% penicillin and streptomycin, at 37°C in a humidified atmosphere containing 5% CO_2_. The cell media was changed every 2 days.

### Liver cancer model

All animal experiments were performed according to protocols approved by the Animal Care and Use Committee of the Medical College of Shantou University. Ten female SD rats (Shantou University Medical Center Laboratory, Shantou, China), weighing 250 to 300 g, were used for this study. SD rats were anesthetized by injecting a solution of chloral hydrate into the enterocoelia. Then, a midline mini-laparotomy was performed and the left lateral hepatic lobe was exposed. McA-RH7777 rat hepatoma cells (1 × 10^7^) were prepared in 0.05 mL of a mixture of serum-free DMEM and Matrigel, and injected into the left lateral lobe [37]. At the end of the operation, the abdominal incisions were closed using a two-layer technique, and tumors were allowed to form for 14 days.

### Breast cancer model

Six MMTV-*Erbb2* transgenic mice were purchased from Jackson Laboratories and bred in-house. This model carries an activated *Erbb2* oncogene driven by the MMTV promoter, leading to the overexpression of constitutively active oncogenic Erbb2 in the breast [38]. These mice develop spontaneous mammary tumors after a latency period and are a good model for human breast cancer.

### MRI

Experiments were performed on a 7T animal MRI scanner (Agilent, VNMRS, USA) at room temperature using a 63 mm internal diameter standard ^1^H volume coil (single-channel). For T_2_ MRI, we used a fast spin echo sequence. The parameters were as follows: relaxation time (TR) = 2000 ms; echo time (TE) = 4.1 ms; number of excitations (NEX) = 1; field of view (FOV) = 50 × 50 mm; slice thickness = 3 mm; matrix = 128 × 128. CEST images were acquired using an echo planner imaging sequence. Continuous wave RF saturation was applied for 5 s, with RF irradiation powers of 1.5, 3, 6, and 9 μT for investigating the CEST Z-spectra of phantoms, and 1.5 and 6 μT for CEST imaging. The other parameters were as follows: TR = 6.0 s; TE = 4.1 ms; NEX = 4; FOV = 30 × 30 mm for *in vitro* and 50 × 50 mm for *in vivo* experiments; slice thickness = 3 mm; matrix = 64 × 64. We obtained Z-spectra ranging from −8 to 8 ppm, at intervals of 0.31 ppm B_0_, and B_1_ field inhomogeneities were mapped and used for removing field inhomogeneity-induced artifacts during the Z-spectra reconstruction [18].

### Animal preparation

During MR imaging, mice were anesthetized by inhaling a mixture of isoflurane (1.5%) and oxygen. Breathing rate was monitored throughout the *in vivo* MRI experiment using a respiratory gating device. Respiratory gating was performed by triggering the start of the pulse sequence soon after the end of the exhalation, with a rate of 40 breaths per min. Ioversol (dose, 4 g I/kg body weight) solution was slowly injected into the tail vein, and we acquired CEST images at two RF power 1.5 and 6.0 μT) 15 min after ioversol injection.

After MRI acquisition, mice were sacrificed and the tumor tissue were excised. Tissue was sectioned and stained with HE for microscopy.

### Statistical analysis

All CEST images were analyzed using MATLAB (The Mathworks, Inc., Natick, MA, USA) [39, 40]. Z-spectra were interpolated by smoothing splines. B_0_-shift correction was defined as the lowest intensity point in the Z-spectrum of each voxel as the central water frequency, then the other data points in the voxel's Z-spectrum were shifted accordingly [34]. Saturation transfer efficiency (ST%) was measured by MTR_asym_ = (S_Δω_ – S_+Δω_) / S_0_, and S_-Δω_, S_+Δω_, with S_0_ representing the water signal with a saturation frequency offset at -Δω, +Δω with and without saturation, respectively. Assessment of the pH_e_ difference between the tumor and normal liver tissue were analyzed using Student's *t*-tests, and a *p* < 0.05 was considered significant.

## SUPPLEMENTARY MATERIALS FIGURES


