# Supplementary material for: Extracellular pH is a biomarker enabling detection of breast cancer and liver cancer using CEST MRI
Source: Oncotarget. 2017 Apr 25;8(28):45759–67. doi: 10.18632/oncotarget.17404 (PMC5542224; doi:10.18632/oncotarget.17404)
Supplement: Supplementary file 1 [file oncotarget-08-45759-s001.pdf]

Extracellular pH is a biomarker enabling detection of breast cancer and liver cancer using CEST MRI

SUPPLEMENTARY INFORMATION

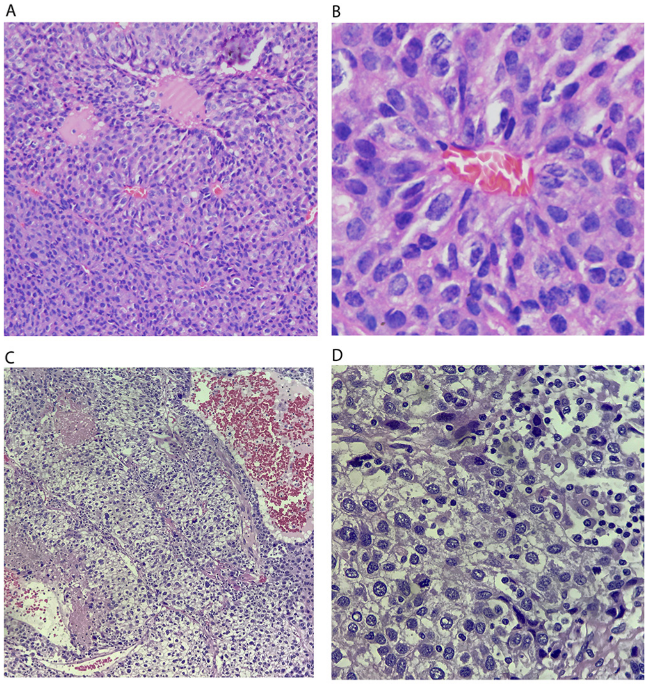

Supplementary Figure 1: Hematoxylin and eosin (H&E) staining. Representative images of breast tumors **(A)** (magnification, ×100) and **(B)** (magnification, ×400). Cancer cells in the central part of the tumor show hydropic cytoplasm degeneration. HE staining of McA-RH7777 tumors shows a similar trabecular histological pattern to that of human hepatocellular carcinoma **(C)** (magnification, ×100) and **(D)** (magnification, ×400).
